# Supplementary material for: One-dimensional carbon chains encapsulated in hollandite
Source: Commun Chem. 2023 Oct 3;6:213. doi: 10.1038/s42004-023-01011-3 (PMC10547843; doi:10.1038/s42004-023-01011-3)
Supplement: Supplementary file 2 — Supplementary Information [file 42004_2023_1011_MOESM2_ESM.pdf]

Supplementary Information

One-Dimensional Carbon Chains Encapsulated in Hollandite

Jonathan M. Polfus[1*]

[1] Department of Chemistry, Centre for Materials Science and Nanotechnology, University of Oslo, PO Box 1033 Blindern, N-0315 Oslo, Norway

[*] Contact e-mail: jonathan.polfus@kjemi.uio.no

Supplementary Methods

DFT calculations were carried out using VASP 6.3 with projector-augmented wave pseudopotentials and the SCAN+rVV10 van der Waals functional.[1-3] The SCAN functional provides reliable description of the structure, electronic properties and thermodynamic stability of MnO_2 polymorphs,[4] and the nonlocal rVV10 density functional accounts for dispersion interactions between the carbon chains and the encapsulating oxide material.[5]

The calculations were spin-polarized with explicit treatment of the following valence electrons: Mn $3s^2p^6d^54s^2$; O $2s^2p^4$; C $2s^2p^2$ and H $1s^1$. The plane wave energy cutoff was 500 eV and the k-point grid was $4 \times 4 \times 16$ grid for the cubic α-MnO_2 unit cell. The convergence criteria were 10^{-6} eV and −0.02 eV Å^{-1} for the electronic structure and atomic positions, respectively. The optimized lattice parameters of α-MnO_2 were a = 9.637 Å and c = 2.835 Å. Antiferromagnetic ordering was imposed with ferromagnetic coupling between two neighboring c-axis columns of edge-sharing manganese octahedra and alternation of these double columns (denoted AFM1).[6,7] Selected simulations were performed with a slightly different antiferromagnetic ordering comprising alternating ferromagnetic columns that run along the c-axis (denoted AFM2).[8] With the current computational parameters, the former configuration was more stable by −0.0007 eV per manganese atom, in complete agreement with the results of Noda et al.[7]

Carbon chains were introduced into one out of two channels in $1 \times 1 \times 4$ supercells of α-MnO_2 (96 atoms). The lattice parameters of the composite cells were relaxed upon encapsulations or fixed to α-MnO_2 whenever specified. Notably, a periodic chain of nine cumulenic carbon atoms shows a minor compressive strain of −1.28% with the relaxed c-axis of the composite supercell (**Supplementary Table 1**), corresponding to a change in the C–C bond length from 1.276 Å to 1.262 Å. The isolated polyenic C_6H_2 molecule was modelled in vacuum using cubic cells of 20 Å and Γ-point sampling. Periodic C_9 was modelled in vacuum with lateral cell parameters of 20 Å and a $1 \times 1 \times 4$ k-point grid. Interaction energies were calculated as the total energy difference between the composite supercell and the isolated materials. The lattice parameters of the composite cell were imposed on the isolated materials in order for the interaction energy to not include the strain induced in both materials due the periodic boundary conditions of the cells.

Carbon nanotube with chiral indices (7,1) and (5,5) were modelled in vacuum with lateral cell parameters of 20 Å and a lengthwise expansion by 2 and 10 unit cells, respectively. The k-point grids were $1 \times 1 \times 2$. The optimized lattice parameters were 20.06 Å for the (7,1) nanotube and 24.56 Å for the (5,5) nanotube, yielding strains of −1.09% and 1.25% for encapsulated C_{17} and C_{19} cumulene chains, respectively.

The activation energy for diffusion of carbon chains along the channels was investigated by the climbing image nudged elastic band (CI-NEB) method[9] as well as static displacement along the channel. Vibrational frequencies and zero-point energies (ZPE) were calculated using the finite displacement method within the harmonic approximation.

Supplementary Table 1: Optimized lattice parameters of cumulenic C_9 and α-MnO_2 (1 × 1 × 4 supercells). Values in parentheses refer to the AFM2 configuration.

Material	Cell parameters	
	a (Å)	c (Å)
C_9	–	11.488
α-MnO_2	9.623 (9.637)	11.326 (11.340)
α-MnO_2 + C_9	9.712 (9.706)	11.341 (11.354)
α-MnO_2 + C_6H_2	9.667 (9.672)	11.329 (11.340)

Supplementary Note 1

Supplementary Table 2 shows the interaction energies between the carbon chains and α-MnO_2 in the AFM2 configuration. The interaction energy between cumulenic C_9 and the encapsulating oxide was similar for both types of antiferromagnetic ordering, i.e., 0.065 eV and 0.056 eV and for AFM1 and AFM2, respectively. The lateral expansion of the composite supercell was 0.13%, and the interaction energy increased to 0.135 eV when relaxation was not allowed, i.e., all cell parameters fixed to α-MnO_2. The C_6H_2 showed a smaller difference in interaction energy between fixed and relaxed lattice parameters, in line with the minor lateral expansion of 0.02% (**Supplementary Table 2**).

Supplementary Table 2: **Interaction energy and structural changes upon encapsulation in α-MnO_2.** The lattice parameters were either fixed to α-MnO_2 or relaxed as given by the lateral expansion. The strain refers to the c-axis of α-MnO_2. The interaction energies are given per carbon atom and the table refers to the AFM1 configuration.

Material	Diameter (Å)	Lateral expansion (%)	Strain (%)		Interaction energy (eV)
			α-MnO_2	C_9	
α-MnO_2 + C_6H_2	4.99	–			0.109
	5.04	0.46			0.090
α-MnO_2 + C_9	4.97	–	–	−1.41	0.135
	5.08	0.92	0.13	−1.28	0.065

Supplementary Figure 1 shows the energy barrier for displacement of cumulene along the α-MnO_2 channel. The obtained barrier of 1.2 meV is miniscule compared to the barrier of about 1 eV for C_6H_2 (see Figure 3 in the main manuscript).

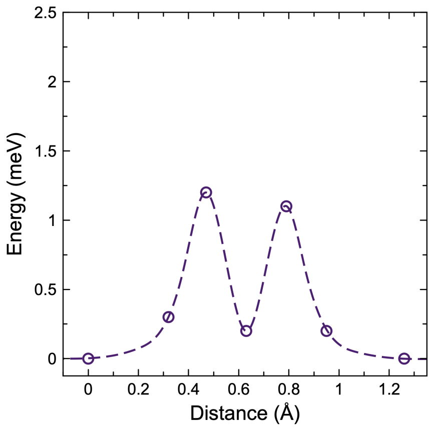

Supplementary Figure 1: Energy barrier for displacement of cumulenic C_9 along the c-axis of α-MnO_2 from CI-NEB. The dashed line is a guide to the eye. The data were obtained with the AFM2 configuration.

Supplementary Figure 2 shows the charge density difference upon encapsulation of C_6H_2. The polarization of the charge densities surrounding the protruding oxide ions is smaller compared to the case with cumulene (see Figure 4 in the main manuscript).

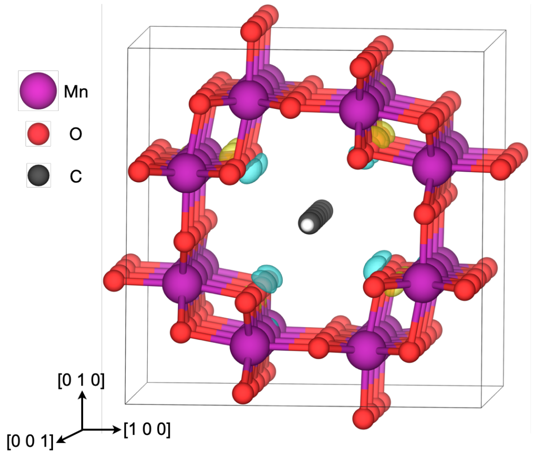

Supplementary Figure 2: Charge density difference upon insertion of C_6H_2 in α-MnO_2. Blue and yellow isosurfaces (0.05 a_0^{-3}) represent negative and positive charge densities, respectively. The data were obtained with the AFM2 configuration.

Supplementary Table 3 shows the vibrational frequencies and zero-point energies of a protruding oxide ion in pristine α-MnO_2 and with encapsulated cumulenic C_9.

Supplementary Table 3: Vibrational frequencies and total zero-point energy of an oxide ion bordering the channel. The change in zero-point energy (ΔZPE) is given relative to the pristine cell . The data were obtained with the AFM2 configuration.

Material	Vibrational frequencies cm^{-1}			ZPE (meV)	ΔZPE (meV)
α-MnO_2	551.3	536.4	530.1	100.29	–
α-MnO_2 + C_9	559.9	538.2	508.9	99.62	−0.67

Supplementary References

1 Kresse, G. & Furthmüller, J. Efficient iterative schemes for ab initio total-energy calculations using a plane-wave basis set. *Phys. Rev. B* **54**, 11169-11186 (1996). https://doi.org/10.1103/PhysRevB.54.11169

2 Sun, J., Ruzsinszky, A. & Perdew, J. P. Strongly Constrained and Appropriately Normed Semilocal Density Functional. *Phys. Rev. Lett.* **115**, 036402 (2015). https://doi.org/10.1103/PhysRevLett.115.036402

3 Peng, H., Yang, Z.-H., Perdew, J. P. & Sun, J. Versatile van der Waals Density Functional Based on a Meta-Generalized Gradient Approximation. *Phys. Rev. X* **6**, 041005-041005 (2016). https://doi.org/10.1103/PhysRevX.6.041005

4 Kitchaev, D. A. *et al.* Energetics of MnO_2 polymorphs in density functional theory. *Phys. Rev. B* **93**, 1-5 (2016). https://doi.org/10.1103/PhysRevB.93.045132

5 Sun, J. *et al.* Accurate first-principles structures and energies of diversely bonded systems from an efficient density functional. *Nat. Chem.* **8**, 831-836 (2016). https://doi.org/10.1038/nchem.2535

6 Crespo, Y. & Seriani, N. Electronic and magnetic properties of α-MnO_2 from *ab initio* calculations. *Phys. Rev. B* **88**, 144428 (2013). https://doi.org/10.1103/PhysRevB.88.144428

7 Noda, Y., Ohno, K. & Nakamura, S. Momentum-dependent band spin splitting in semiconducting MnO_2: a density functional calculation. *Phys. Chem. Chem. Phys.* **18**, 13294-13303 (2016). https://doi.org/10.1039/c5cp07806g

8 Kitchaev, D. A., Dacek, S. T., Sun, W. & Ceder, G. Thermodynamics of Phase Selection in MnO_2 Framework Structures through Alkali Intercalation and Hydration. *J. Am. Chem. Soc.* **139**, 2672-2681 (2017). https://doi.org/10.1021/jacs.6b11301

9 Henkelman, G., Uberuaga, B. P. & Jónsson, H. A climbing image nudged elastic band method for finding saddle points and minimum energy paths. *J. Chem. Phys.* **113**, 9901-9904 (2000). https://doi.org/10.1063/1.1329672
